# Hyaluronic Acid-Mediated Drug Delivery System Targeting for Inflammatory Skin Diseases: A Mini Review

**DOI:** 10.3389/fphar.2020.01105

**Published:** 2020-07-24

**Authors:** Kang Nien How, Wei Hsum Yap, Calvin Lai Hock Lim, Bey Hing Goh, Zee Wei Lai

**Affiliations:** ^1^ Dermatology Unit, Faculty of Medicine and Health Sciences, Universiti Putra Malaysia, Serdang, Malaysia; ^2^ Faculty of Medical and Health Sciences, School of Biosciences, Taylor’s University, Subang Jaya, Malaysia; ^3^ College of Pharmaceutical Sciences, Zhejiang University, Hangzhou, China; ^4^ Biofunctional Molecule Exploratory Research Group (BMEX), School of Pharmacy, Monash University Malaysia, Bandar Sunway, Malaysia

**Keywords:** hyaluronic acid, inflammatory skin diseases, psoriasis, atopic dermatitis, drug delivery, CD44 receptor

## Abstract

Hyaluronic acid (HA), a major component of extracellular matrix has been widely applied in pharmaceutical and cosmetic industries due to its reported pharmacological properties. Various types of HA drug delivery system including nanoparticles, cryogel-based formulations, microneedle patches, and nano-emulsions were developed. There are studies reporting that several HA-based transdermal delivery systems exhibit excellent biocompatibility, enhanced permeability and efficient localized release of anti-psoriasis drugs and have shown to inhibit psoriasis-associated skin inflammation. Similarly HA is found in abundant at epidermis of atopic dermatitis (AD) suggesting its role in atopic AD pathology. Anti-allergenic effect of atopic eczema can be achieved through the inhibition of CD44 and protein kinase C alpha (PKCα) interaction by HA. Herein, we aim to evaluate the current innovation on HA drug delivery system and the other potential applications of HA in inflammatory skin diseases, focusing on atopic dermatitis and psoriasis. HA is typically integrated into different delivery systems including nanoparticles, liposomes, ethosomes and microneedle patches in supporting drug penetration through the stratum corneum layer of the skin. For instance, ethosomes and microneedle delivery system such as curcumin-loaded HA-modified ethosomes were developed to enhance skin retention and delivery of curcumin to CD44-expressing psoriatic cells whereas methotrexate-loaded HA-based microneedle was shown to enhance skin penetration of methotrexate to alleviate psoriasis-like skin inflammation. HA-based nanoparticles and pluronic F-127 based dual responsive (pH/temperature) hydrogels had been described to enhance drug permeation through and into the intact skin for AD treatment.

**Graphical Abstract:**
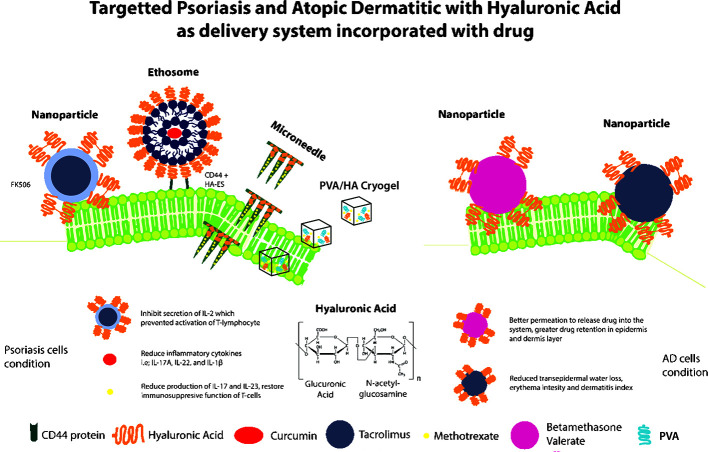
HA-mediated Drug Delivery System Targeting Inflammatory Skin Diseases (Psoriasis and Atopic Dermatitis).

## Introduction

Skin as the first line of defense against infection is made up of three main layers which vary in functions and anatomy: epidermis (outer layer), dermis (middle layer) and hypodermis (bottom layer) (Prost-Squarcioni, 2006). As it interacts with environment, skin acts as a protector against diseases, excessive water loss, UV damage, mechanical injury, and also helps in regulate body temperature (Logger et al., 2019). Skin and subcutaneous diseases are the 4th leading cause of disability globally in 2013 excluding mortality, with dermatitis being the greatest burden among all costing approximately 9.3 million disability-adjusted life years (Karimkhani et al., 2017). Psoriasis is reported to affect 2% of worldwide population due to genetic and environmental factors (Mohd Affandi et al., 2018). Psoriasis is an inflammatory skin diseases which is chronic with strong genetic predisposition and autoimmune pathogenic traits (Du et al., 2019) that is causing burden to patient either physically or physiologically (Boehncke and Schön, 2015). For mild to moderate psoriasis, topical corticosteroids are the first-line treatment. However, long term application of topical corticosteroids can produce adverse effects such as epidermal thinning, atrophy, ulceration and facial erythema (Coondoo et al., 2014).

Atopic dermatitis (AD), a common chronic inflammatory skin disease poses a significant threat to both patient and the economy. It also affects the quality of individual life as well as their families (Kapur et al., 2018). The disease is believed to arise due to the complex interplay between skin barrier malfunction, immune dysregulation and infectious agents from environment factor (Kapur et al., 2018). In Canada, it was reported that an estimated cost of $1.4 billion annually is incurred to assist AD patient in getting treatment, like emollients application, as well as frequent physician visits which impacted the economy (Barbeau and Lalonde, 2006). AD has significant impact on the patients quality of life as there will be constant itchiness which can lead to skin trauma and sleep deprivation (Kapur et al., 2018).

CD44, a receptor for HA is found to be highly expressed in psoriatic skin. The concentration is negatively correlated with HA distribution (Zhang et al., 2019). It was found that anti-allergenic effect can be achieved through the inhibition of CD44 and PKCα interaction by HA (Kim et al., 2008). Based on the understanding of the characteristic of HA and HA-CD44 pathway, HA is widely used as a nano-drug delivery system to enhance the therapeutic effect of drug at the lesion site (Taetz et al., 2009). HA assisted drug delivery systems that has been described include nanoparticle, ethosomes and microneedle. It is an important vehicle to enhance drug-skin penetration and an important vehicle to retain moisture in AD in view of barrier dysfunction. The objective of this review is to review the role of HA as a drug delivery system in inflammatory skin diseases namely psoriasis and atopic dermatitis.

## Structural and Functional Characteristics of Hyaluronic Acid

Hyaluronic acid (HA), a major component of extracellular matrix has been widely applied in pharmaceutical and cosmetic industries due to its reported pharmacological properties which includes anti-aging (Bukhari et al., 2018), anti-inflammatory (Chen, 2018), skin repairing (Narurkar et al., 2016), tissue regeneration (Bukhari et al., 2018) and wound recovery properties (Chen, 2018). HA possess good biocompatibility, high moisture retention, and tuneable viscoelastic properties (Sze et al., 2016). It is a natural unbranched polymer composed of repeating disaccharide units of D-glucuronic acid and N-acetylglucosamine linked by a glucuronidic β (1–3) bond. HA with different molecular weights exhibit distinct properties. For instance, low molecular weight HA (LMW-HA) is reported to be associated with promoting angiogenesis whereas high molecular weight HA (HMW-HA) inhibits angiogenesis. The appropriate balance between synthesis and degradation of HA is vital in the regulation of various biological functions including cell proliferation, migration, differentiation (Bychkov and Kuzmina, 2015), vasculogenesis and angiogenesis (Pardue et al., 2008), as well as regulating cell adhesion and motility (Kouvidi et al., 2011), as determined by their molecular weight. LMW-HA on the other hand exerts opposite effect as compared to HMW-HA where it demonstrates biological activities such as antioxidant properties and pronounced free radical scavenging has been developed in recent years (Ke et al., 2011). In addition, LMW-HA has also shown to induce inflammatory genes in T-24 carcinoma cells, in eosinophils (Ohkawara et al., 2000) as well as dendritic cells (Termeer et al., 2000). Both sizes of HA are safe and efficacious, and the market is expecting a continuing emergence of HA-based products in the coming years (Tabassum and Hamdani, 2014).

## Psoriasis

Psoriasis is a chronic inflammatory autoimmune skin disease characterized by marked epidermal proliferation and abnormal differentiation with activation of both innate and acquired immunity (Rendon and Schäkel, 2019). This autoimmune disorder is multifactorial, and inflammation is known to play a major role in its development. Research have showed that activated Th1 and Th17 T cells (CD4+ T cells), CD8+ T cells, as well as increased levels of cytokines such as IL-17, IL-23, TNF-α and IL-27, have been directly implicated in psoriasis immunopathogenesis (Luger and Loser, 2018). The immunology and genetics studies of psoriasis have provided insights into the heterogeneity and regulatory pathways that govern psoriasis, and assist in the development of therapy based on patients biogenetic markers and identify new avenues for treatment based on a more complete understanding of the immunological mechanisms. Topical administration is one of the important approaches to treat psoriasis. Drugs are applied directly to the affected skin lesions to inhibit inflammatory symptoms of psoriasis. Topical administration of drugs with narrow therapeutic window can reduce systemic absorption compared to drugs delivered through oral and intravenous routes which help to reduce adverse systemic effects. Though topically applied drugs provide prolonged duration of action, the stratum corneum limits the amount of drugs being percutaneously absorbed, resulting in poor clinical efficacy. Hence, novel and improved percutaneous delivery system to overcome the stratum corneum barrier of psoriatic skin is vital to antipsoriatic drug for topical therapy.

## HA as a Drug Delivery System in Psoriasis

Currently, HA is widely incorporated as part of the transdermal delivery system to enhance drug penetration in psoriasis treatment. Various forms of drug delivery systems including HA-based nanoparticles, ethosomes, cryogels, and microneedle patches have been developed to enhance drug permeation through and into the intact skin for psoriasis treatment. The excellent solubility properties of HA resulted in its development as one of the most important topical carriers for the localized delivery of drugs to the skin and also as a drug delivery agent for ophthalmic, nasal, pulmonary, parenteral and topical routes of administration (Brown and Jones, 2005). HA acts as mucoadhesive, retaining the drug at specific site of action/absorption. It also can modify the *in vivo* release and absorption rate of the therapeutic agent and it is able to localize delivery of drug to the epidermis. The types of psoriasis drugs that have been incorporated into these HA-based delivery systems include methotrexate, tacrolimus, and corticosteroids, all which are first-line treatments for moderate to severe psoriasis.

One of the novel nano-topical drug delivery system developed using HA-modified ethosomes target CD44 in the inflamed epidermis (Zhang et al., 2019). Ethosomes are novel deformable liposomes derived from dispersing liposomes in small-chain biocompatible alcohols and they are demonstrated to be more superior than classic liposomes which significantly increase skin retention of drugs (Touitou et al., 2000). The incorporation of curcumin in HA-modified ethosomes target CD44 in the inflamed epidermis (Zhang et al., 2019). Recent studies have found that CD44 protein is highly expressed in the epidermis of psoriatic inflamed skin, suggesting that CD44 can serve as a potential target of novel active-targeting nanocarriers for topical administration to increase skin drug retention and enhance drug efficacy (Lindqvist et al., 2012). The HA-modified ethosomes showed specific adhesion CD44 in imiquimod-induced psoriasis-like inflamed skin, and that the increased topical drug delivery of curcumin reduced TNF-α, IL-17A, IL-17F, IL-22, and IL-1β mRNA levels; and lower CCR6 protein expression.

Another formulated hybrid nanoparticle system based on the combination of amphiphilic conjugations of HA–Cholesterol-self-assembled nanoparticles and hydrotropic nicotinamide was developed to enhance the permeation of tacrolimus (FK506) in the treatment of psoriasis (Wan et al., 2017). Commercial FK506 is an ointment formulation use to treat atopic dermatitis, but it is applied to other skin diseases like psoriasis (Malecic and Young, 2016). FK506 is a type of macrolide immunosuppressive drug which prevents activation of T-lymphocyte through inhibition of IL-2, a cytokine that is responsible for the development and persistence of psoriasis lesions (Goebel et al., 2011). However, topical treatment of tacrolimus ointment on hyperkeratotic psoriatic plaques did not promote FK506 deposition due to its high hydrophobicity and high molecular weight (Zonnevdd et al., 1998). The combination of HA–Cholesterol nanoparticles exhibited a significant synergistic effect on the permeation of FK506 ointment and that it presented a synergistic effect on antipsoriasis which might increase the therapeutic effect and minimize systemic side effects (Wan et al., 2017).

Synthetic biodegradable polymers have significant versatility and diverse biomedical applications owing to their tailorable designs or modifications. Poly(vinyl alcohol) (PVA) is a water soluble biodegradable synthetic polymer with good biocompatibility, and it can be physically cross-linked by the freeze-thaw method (Stauffer and Peppast, 1992) to form hydrogels useful in pharmaceutical formulations. The development of PVA/HA cryogels loaded with methotrexate showed good *in vivo* biocompatibility after systemic and topical administration in laboratory animals. The pH-responsive swelling and releasing abilities of these PVA/HA cryogels allow these systems to be developed as topical formulations for psoriasis therapy.

Meanwhile, development of pH-responsive biodegradable poly-L-glutamic acid (PGA)-fluocinolone acetonide (FLUO) conjugate allows the controlled release of the FLUO to reduce skin inflammation (Dolz-Pérez et al., 2020). However, PGA-FLUO showed limitations in the release of drug in the epidermis which bring about the application of PGA-FLUO within hyaluronic acid (HA)-poly-L-glutamate cross polymer (HA-CP) resulted in slower and sustained drug transfer in the epidermis allowing sufficient residence time for drug activity (Dolz-Pérez et al., 2020).

Microneedle patch is a highly efficient and versatile device which attracted extensive scientific and industrial interests in the past decades due to prominent properties including painless penetration, low cost, excellent therapeutic efficacy, and relative safety. The robust microneedle enabling transdermal delivery has a paramount potential to create advanced functional devices with superior nature for biomedical applications. Methotrexate, an analogue of folic acid has been used as a drug to treat psoriasis skin condition (Czarnecka-Operacz and Sadowska-Przytocka, 2014). Methotrexate has been shown to down-regulate interleukin-17 and interferon-c, inhibit proliferation of effector T cells and restore the immunosuppressive function of regulatory T cells (Chen, 2018). Nevertheless, its high molecular weight, hydrophilicity and inability to maintain a stable form at physiological pH restricted the permeability of methotrexate by passive diffusion through the stratum corneum (Alvarez-Figueroa et al., 2001). The development of methotrexate-loaded HA-based microneedle patch successfully penetrated imiquimod (IMQ)-induced thickened epidermis in mice and alleviated the psoriasis-like skin inflammation (Du et al., 2019). In addition, the methotrexate-loaded HA-based microneedle patches were significantly more efficacious than taking the same dose of drug orally. This significantly reduced the amount of methotrexate required for the microneedle patch delivery system required for a comparable amelioration, which in turn lowered its systemic toxicity (Du et al., 2019).

## HA as a Biomarker in Psoriasis

Serum HA had been utilized as a biomarker for psoriasis as studies reported that concentration of serum HA increases significantly in patients with serious cutaneous psoriasis (Yamamoto et al., 1997). This is due to the ability of low molecular weight HA (LMWHA) in inducing inflammatory cytokine gene expression and serving as a ligand for toll-like receptors (TLRs). In addition, presence of IL-1β, tumor necrosis factor-α (TNF-α) and degradative enzymes such as metalloproteinases will lead to an increase in hyaluronan synthetases (HAS) 1–3 and hyaluronidases that synthesize LMWHA by degrading HMWHA (Mehta et al., 2011; Hellman et al., 2019). In psoriasis, TLRs play a vital role as innate immune responses through activation of TLR7 and TLR9 and also the secretion of interleukin-23 (IL-23) *via* autoimmune plasmacytoid dendritic cell activation which releases interferon-a, that further signals Tip-DC (tumor necrosis factor-a and inducible nitric oxide synthase-producing dendritic cell) activation (Yamamoto, 2015). Other TLRs including TLR2 and TLR4 contribute to the pathogenesis of psoriasis plaque, guttate (Garcia-Rodriguez et al., 2013) and also psoriatic arthritis (Carrasco et al., 2011). HA is highly abundant in psoriatic skin, and it signals through TLR4 and TLR2. Meanwhile the binding of HA on CD44 also induces secretion of IL-6 and inflammation in T cells and neutrophils (Jiang et al., 2011).

## Atopic Dermatitis

Atopic dermatitis (AD) is the most common chronic inflammatory skin disease affecting infants and children (Siegfried and Hebert, 2015). Factors causing AD includes defect in terminal epithelial differentiation, immune dysregulation, altered skin microbiome and altered composition of stratum corneum (SC) intracellular lipids leading to barrier dysfunction (Bieber et al., 2017; Nakatsuji et al., 2017). Impairment of the SC permeability barrier functions cause dehydration due to an increase of transepidermal water loss, followed by inflammation due to the increasing release of cytokines, chemokines and interleukines (Damiani et al., 2019). Skin hydration, anti-bacterial measures and topical immunosuppressant therapy using topical corticosteroids calcineurin inhibitors are the common approaches in the management of AD (Sathishkumar and Moss, 2016).

## HA’s Role in Atopic Dermatitis

HA is found to be related to inflammatory skin dermatoses (Ohtani et al., 2009; Barnes et al., 2012) The synthesis of HA is regulated by hyaluronan synthases (HAS). In normal circumstances, HAS1 is found to be regulating epidermal differentiation. However, role of HAS3 becomes apparent during pathological condition (Malaisse et al., 2014). The earlier studies provide conflicting evidences on HA role in epidermal differentiation (Maytin et al., 2004; Passi et al., 2004; Bourguignon et al., 2006; Farwick et al., 2011). Latest evidence by Malaisse et al. (2014) acknowledge the fact that though HA present at high level, it is not necessary for homeostatic epidermal differentiation (Malaisse et al., 2014). However, HA become important in pathological condition. During inflammation, HAS3 is upregulated while HAS1 is downregulated. Heparin binding- epidermal growth factor. (HB-EGF) is found to be possibly linked to HAS expression in AD lesions. However, how HAS involved in the pathomechanism of keratinocyte inflammation is still unclear and thus requiring further studies (Malaisse et al., 2014).

## HA in Medical Device Moisturizers

The basic principle AD treatment is optimal skin care that address the skin barrier defect. Moisturizers restore the ability of lipid bilayer to restore, retain and redistribute water. They penetrate and contribute to the reorganization of the structure of the skin layers. Thus, moisturizers play an important role in AD management (Giam et al., 2016). Moisturizers can be categorized into three main groups, namely humectant, occlusive and emollients. These products are formulated in various delivery system including gel, foam, cream, ointment and lotion. They also contain various composition to enhance its efficacy. Humectants work by attracting fluids from the deeper epidermis to SC and has a biological similarities to the natural moisturizing factors of the corneocytes (Giam et al., 2016). HA being a natural component of the skin, has been one of the most favorite humectant used in almost all nutricosmetic products with moisturizing properties (Bukhari et al., 2018). It helps in attracting and retaining fluid, and skin barrier repair (Maytin et al., 2004; Goh et al., 2019). However it is rarely used primarily as a sole ingredient, but rather working synergistically with other category of moisturizer to achieve its role in skin repair (Draelos, 2011; Frankel et al., 2011; Pacha and Hebert, 2012; Micali et al., 2018). The molecular weight of HA shall be considered, as HA with a molecular weight higher than 50 kDa may reduce skin penetration. Thus, HMWHA is used to form a film to impede epidermal water loss, while LMWHA is utilized to improve skin penetration to restore a sustained physiological and hydrated microenvironment for optimize skin rejuvenation and tissue repair (Symonette et al., 2014).

## HA as a Drug Delivery System in Atopic Dermatitis

Similar to psoriasis, various forms of drug delivery systems including HA-based nanoparticles and pluronic F-127 based dual responsive (pH/temperature) hydrogels have been described to enhance drug permeation through and into the intact skin for AD treatment. The types of drugs that have been incorporated into these HA-based delivery systems include tacrolimus, bethamethasone valerate and gallic acid, a principal component of traditional Chinese medicine Cortex Moutan.

Topical calcineurin inhibitor, namely tacrolimus ointment (Protropic^®^) and pimecrolimus cream (Elidel^®^) are steroid sparing agents for the treatment of atopic dermatitis. However the potency of tacrolimus 0.1% was only found to be equivalent to medium potency topical steroid(Eichenfield et al., 2014), possibly due to the limited skin penetration due to its large molecular size. A novel HA-coated tacrolimus-loaded nanoparticles (HA-TCS-CS-NPs) was described (Zhuo et al., 2018). It was noted that the physiochemical properties had been optimized, *in vivo* drug release was more sustained, drug permeation and retention had been improvised, and anti-AD efficacy in AD mice model was found to be superior in comparison with tacrolimus solution (Zhuo et al., 2018). However, this study did not compare their nanoparticles with the marketed Protropic^®^. Similar technology had been used to encapsulate betamethasone valerate (BMV), a medium potency topical corticosteroid. It was found that (HA-BMV-CS-NP) demonstrated optimum physicochemical characteristics, including fine particle size, high zeta potential, high entrapment efficacy and loading capacity. It also demonstrated higher drug permeation and drug retention(Pandey et al., 2019).

Pluronic F-127 (PF127) is a non-ionic triblock copolymer of poly(ethylene oxide)-b-poly(propylene oxide)-b-poly(ethylene oxide), thermo-responsive polymer capable to form gel from its aqueous solution. Chitosan is a cationic pH-responsive biopolymer that swells in acidic pH and shrinks in basic pH, while HA is an anionic pH-responsive biopolymer that can swell in basic pH and shrink in acidic pH (Chatterjee et al., 2019). The conjugation of these polymers formed a dual-responsive hydrogels [PF127/HA-Ala-Chito(oligo)]. It had been used to incorporate gallic acid, the principal component of traditional Chinese medicine Cortex Moutan, recommended in the treatment of atopic dermatitis. This anti-inflammatory and anti-allergic herbal medicine is claimed to have similar effect like corticosteroids without their side effects. PF127/HA-Ala-chito(oligo) drug delivery system was found to have improve rheological properties, mechanical stability and pH responsiveness. This drug delivery system was found to exhibit better gallic acid release under neutral pH condition. The mechanical stability and sustain drug release property despite increasing acidity in the external environment. However, further modification of this drug delivery system is required to involve non-toxic biomaterials (Chatterjee et al., 2019).

## Conclusion

HA is widely utilized in dermatology as it is a natural, non-immunogenic polysaccharide with good biocompatibility and degradability (Huang and Huang, 2018). The moisturizing properties of HA made it exist in almost all moisturizing and antiaging cosmetics. The identification of CD44 receptors and HA on both psoriasis and AD epidermis had led to many researchers exploring this component as a possible new way for drug delivery Research on this field flourishes further even more with the improve understanding of nanomedicine. However, the current research are mainly in the pre-clinical phase. There are still large gaps in terms of best material to be utilized to improve the safety and efficacy of drug delivery. The mechanism of HA based drug delivery system is still unclear. The role of HA and its receptor on both AD and psoriasis is also yet to be well defined. Thus, more work needs to be done on all aspect of HA and its receptors on patho-mechanism of inflammatory skin diseases and its potential role in improvising future drug delivery.

## Author Contributions

KNH, ZWL, and WHY conceived the idea and wrote the manuscript. All authors contributed to the article and approved the submitted version.

## Funding

This work was supported by the Ministry of Education (MOE) Fundamental Research Grant Scheme (FRGS/1/2019/STG05/TAYLOR/03/3) awarded to ZWL and (FRGS/1/2019/SKK08/TAYLOR/02/2) awarded to WHY.

## Conflict of Interest

The authors declare that the research was conducted in the absence of any commercial or financial relationships that could be construed as a potential conflict of interest.

## Abbreviations

HA, hyaluronic acid; AD, atopic dermatitis; LMWHA, low molecular weight hyaluronic acid; HMWHA, high molecular weight hyaluronic acid; IL, interleukin; SC, stratum corneum; PKCα; protein kinase C alpha; HaCat, human keratinocyte cell line.
